# Enhanced* Ex Vivo* Expansion of Human Hematopoietic Progenitors on Native and Spin Coated Acellular Matrices Prepared from Bone Marrow Stromal Cells

**DOI:** 10.1155/2016/7231567

**Published:** 2016-02-14

**Authors:** Samiksha Wasnik, Suma Kantipudi, Mark A. Kirkland, Gopal Pande

**Affiliations:** ^1^CSIR-Centre for Cellular and Molecular Biology, Uppal Road, Hyderabad 500 007, India; ^2^Deakin University, Waurn Ponds, Geelong, VIC 3220, Australia; ^3^Sridevi Maternity and Nursing Home, Warasiguda, Hyderabad 500 361, India

## Abstract

The extracellular microenvironment in bone marrow (BM) is known to regulate the growth and differentiation of hematopoietic stem and progenitor cells (HSPC). We have developed cell-free matrices from a BM stromal cell line (HS-5), which can be used as substrates either in native form or as tissue engineered coatings, for the enhanced* ex vivo* expansion of umbilical cord blood (UCB) derived HSPC. The physicochemical properties (surface roughness, thickness, and uniformity) of native and spin coated acellular matrices (ACM) were studied using scanning and atomic force microscopy (SEM and AFM). Lineage-specific expansion of HSPC, grown on these substrates, was evaluated by immunophenotypic (flow cytometry) and functional (colony forming) assays. Our results show that the most efficient expansion of lineage-specific HSPC occurred on spin coated ACM. Our method provides an improved protocol for* ex vivo* HSPC expansion and it offers a system to study the* in vivo* roles of specific molecules in the hematopoietic niche that influence HSPC expansion.

## 1. Introduction

Human stromal cells, residing in hematopoietic niches in the BM, provide cellular and molecular signals that are essential for the regulation of hematopoiesis [1]. Many tissue engineered biological and nonbiological scaffolds have been used for* ex vivo* expansion of HSPC. Bioengineered 2D and 3D scaffolds, which mimic the* in vivo* functional properties of hematopoietic niches, are gaining importance in the research and clinical trials [2]. These scaffolds mainly comprise ACM that are prepared by decellularizing either the whole tissue [3], organ, or cultured stromal cells [4]. The ACM comprises specific extracellular matrix (ECM) components, such as fibrous and nonfibrous collagen, fibronectin or laminin, glycosaminoglycans (GAGs), proteoglycans, growth factors, and cytokines [5]. These components individually or collectively provide biological cues that regulate proliferation, differentiation, and survival of stem and progenitors cells of different types* in vivo* [6]. Some cell types that have been expanded on ACM-based substrates include mesenchymal stem cells (MSCs) [7], neuronal cells [8], osteocytes [9], and embryonic stem cell-derived hepatic cells [10].

A recent study has shown that substrates containing a cocktail of fibronectin, heparin sulphate, tropocollagen I, hyaluronic acid, and cofibrils of collagen I bound to heparin or hyaluronic acid can be used for the adhesion, proliferation, and differentiation of human HSPC [11]. A few earlier studies have used these matrix components individually for the same purpose [12, 13]. However, utilization of the entire ACM as substrates or scaffolds for* ex vivo* lineage-specific HSPC expansion has been limited.

We have previously shown that ACM, prepared from a mouse BM stromal cell line (MS-5), can mimic the endosteal and/or vascular niche-like properties of human BM and support* ex vivo* expansion of UCB HSPC [4]. Our results showed that MS-5 cell line derived ACM could support a significant level (up to 80-fold) of common myeloid progenitor (CMP) cell expansion [4]. In order to develop a more clinically relevant nonxenogeneic model, we report here the potential of ACM generated by human BM stromal cell line (HS-5) on supporting HSPC expansion. HS-5 cells have a fibroblast-like morphology and secrete significant levels of various cytokines, such as granulocyte colony-stimulating factor (G-CSF), granulocyte-macrophage-CSF (GM-CSF), macrophage-CSF (M-CSF), Kit Ligand (KL), macrophage-inhibitory protein-1 alpha, interleukin-6, interleukin-8, and interleukin-11 (IL-6, IL-8, and IL-11) into the culture media [14]. Intact HS-5 cells and their conditioned media have been shown to support the proliferation of HSPC in serum-deprived conditions [14]. For clinical use, it would be preferable to be able to manufacture a standardised ACM product, and we have therefore compared the effects of native HS-5 ACM to a sonicated and solubilised ACM preparation that can be spin-coated onto cell culture surfaces.

## 2. Materials and Methods

### 2.1. Preparation of CD34^+^ Cells

UCB samples were collected from full-term delivery with informed and signed consents following the local ethical guidelines of the institution. Each cord blood sample was collected, by the trained clinicians, into the blood collection bags containing 49 mL of citrate phosphate dextrose anticoagulant (CPDA). Standard protocols were followed for isolation of the target cell (CD34^+^) population from UCB for multilineage* ex vivo* expansion using the previously reported method [15]. Briefly, the UCB was processed by density gradient centrifugation using Ficoll Paque (GE Healthcare Bio-Sciences, Sweden) to obtain the total mononuclear cells (MNCs). CD34^+^ cells were isolated by using CD34 MicroBead Kit, in AutoMACS (Miltenyi Biotec Inc., Germany) by the magnetic assisted cell sorting (MACS) method. Observed cell enrichment was more than 90%. The percentage purity of the MACS-sorted cells was determined by a preexpansion flow-cytometry assay for CD34^+^CD45^lo^, CD34^+^CD38^−^, and CD34^+^CD133^+^ cells surface markers that represent different levels of HSPC differentiation. The isolated UCB CD34^+^ cells were further cultured on ACM substrates. Phenotypic analysis of expanded cells was performed by flow cytometry and functional analysis by colony forming unit assays.

### 2.2. HS-5 Cell Culture

#### 2.2.1. HS-5 Culture Maintenance

HS-5 cell line was obtained from American Type Culture Collection (ATCC, Cat. number CRL-11882). HS-5 cells were cultured in complete growth medium containing *α*-MEM, 10% FBS, 1% Pen/Strep, and 1% GlutaMAX. Cultured cells were maintained in 5% CO_2_, 20% O_2_, and 90% humidity in standard cell culture incubators with regular media change. The cells were grown until they were 80–90% confluent before splitting to the next passage.

#### 2.2.2. HS-5 Cells for HSPC Expansion

HS-5 cells were maintained in complete growth medium as discussed above. After the cells reached 80% confluency, they were shifted to a media containing 1% FBS to limit the mitotic activity of the cells. These mitotically inactive cells were used as cellular control to expand UCB derived HSPC. All experiments were performed with cells maintained within 4–6 passages. Blank tissue culture plates (TCP) without any matrix or cells were used as negative control throughout all the expansion and matrix characterization experiments.

### 2.3. ACM Preparation

#### 2.3.1. Native ACM

**Figure 1:**
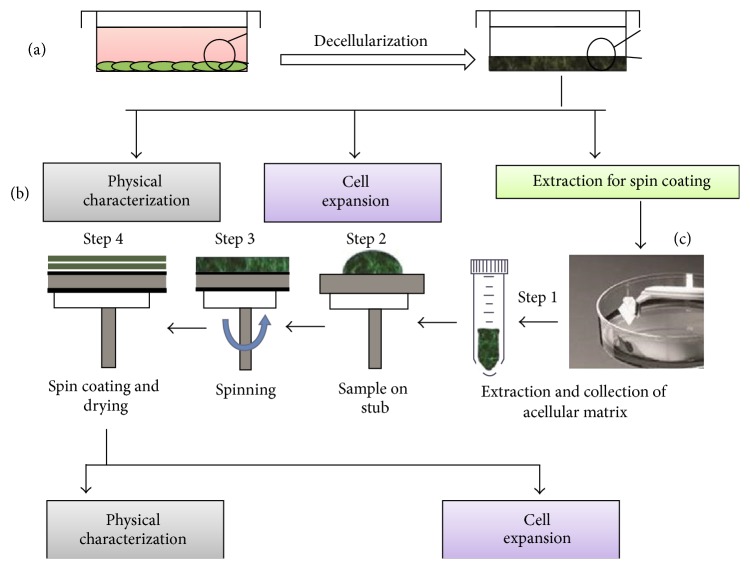
Preparation of spin coated HS-5 acellular matrices. (a) Decellularization of HS-5 cells. (b) Physical characterization of the acellular matrices. (c) Spin coating process: step 1—extraction and collection of acellular matrices, step 2—sample kept on tissue culture surfaces to be coated, which is held in vacuum on a stub, step 3—spinning at fixed rpm, and step 4—coating and drying under sterile conditions.

Native ACM was prepared by decellularization of HS-5 cells. Cells were maintained in complete growth medium for cellular control as described in Section 2.2.2. Before decellularization, cells were further grown for additional 4 days after confluency for better ECM deposition. All procedures for decellularization were done under sterile conditions as mentioned elsewhere [15] (summarised in Figure 1). Briefly, equal numbers of HS-5 cells grown on 24-well plates (Becton Dickinson Biosciences Labwares, USA) were washed with phosphate buffer saline (PBS) and then incubated in MilliQ water until the shape of cells became round. The cells were treated with 0.02 M NH_4_OH and the residual cellular matrices were retained on the surface. ACM was dried and washed with PBS to remove traces of NH_4_OH. The ACM obtained on 24-well plates were used as native ACM substrates.

#### 2.3.2. Spin Coated ACM

For spin coating, ACM suspensions obtained from 60 mm dishes were pooled and briefly sonicated and total protein content was estimated. The fine matrix suspension was immediately processed for spin coating the 24-well tissue culture plates (TCP) for expansion purpose. Based on the total estimated matrix protein in each well of 24-well plate (see Supplementary Table 1 of the Supplementary Material available online at http://dx.doi.org/10.1155/2016/7231567), equal amounts of ACM (~50 *μ*g protein) were placed on each well of a 24-well TCP or borosilicate glass coverslips (18 × 18 mm) and spin coated for 60 sec at 1000 rpm under vacuum, using a WS-400 6NPP-Lite Spin Coater (Laurell Technologies Corporation, USA) under sterile conditions (Figure 1). The process was repeated three times to get maximum layering. The coated plates and coverslips were dried overnight in the laminar flow cabinet, washed in PBS with Pen/Strep, and used for* ex vivo *HSPC expansion and ACM characterization process. Each expansion experiment was done in freshly prepared and spin coated ACM substrate. The amount of protein coated and the parameters of spin coating were kept standard for each experiment.

### 2.4. ACM Characterization

#### 2.4.1. Phase Contrast Microscopy

HS-5 stromal cells and native ACM were incubated with 4% formaldehyde for 20 min at room temperature (RT) followed by washing with 1x PBS. Fixed cells and matrices were observed under light microscope (Axioplan 2, Carl Zeiss, Germany). Image acquisition and processing was done using AxioVision software (Version Release 4.4, Carl Zeiss, Germany).

#### 2.4.2. Scanning Electron Microscopy

Morphological characterization of the native and spin coated ACM was performed by SEM. Both substrates were rinsed in PBS, fixed in 2.5% glutaraldehyde, washed and dehydrated in increasing concentrations of ethyl alcohol (50%, 60%, 70%, 80%, 90%, and 100%), and finally kept in acetone. The fixed and dehydrated samples were then subjected to critical point drying (CPD) and sputter coating with a thin layer of gold. The samples were examined at a range of 5–10 kV in the microscope (3400N, Hitachi, Japan).

#### 2.4.3. Atomic Force Microscopy

Freshly prepared native and spin coated ACM was dried in a dehumidified desiccator for 16 h and subjected to AFM measurements. AFM analysis was carried out in 200–300 kHz tapping modes using a multiprobe imaging microscope Multiview 4000*™* (Nanonics Imaging Ltd., Israel) equipped with a hybrid scanner. Images were collected at scanning frequencies of 5 kHz using glass tip cantilevers at 512 × 512 pixels resolution. Scan regions of 5 × 5 or 2 × 2 *μ*m^2^ were used for data collection and data of multiple scan regions were gathered and averaged (as indicated in Section 3). Root-mean-square values were used to calculate average surface roughness. For calculation of the coating thickness, a scratch on the native or spin coated surface was made to expose the underlying plastic or glass surface and differences in the data values of the scratched and exposed surfaces were used to calculate the thickness of the matrix. For final image reconstruction and calculation of height variations, thickness, and surface roughness, software provided by the manufacturers (WSxM v4.3) was used.

### 2.5. Cytochemical Characterization

Various cytochemical stains were used to stain the native and spin coated ACM for detection of major ECM components. The presence of collagenous matrix in the native and spin coated ACM was demonstrated by staining with Masson's Trichrome kit (Sigma, USA) as instructed by the manufacturer. Similarly, fibrillar collagen was detected by staining the native and spin coated ACM with Picro Sirius Red. Proteoglycans and ECM-bound GAGs were detected by staining with Safranin-O and Alcian Blue, respectively. Details of each of these staining protocols have been described in the supplementary information section. 

### 2.6. Immunofluorescence Characterization

Immunofluorescence staining was performed to examine the expression of major ECM proteins in native and spin coated ACM. Samples were fixed in 4% formaldehyde for 20 min at RT followed by blocking in 3% BSA for 1 hr. The fixed native and spin coated ACM samples were incubated with the appropriate dilution of Phalloidin actin Alexa Flour-488 (Molecular Probes, Invitrogen, USA) in 1% BSA in PBS for 45 min in dark at RT. All the antibodies used in immunofluorescence studies were purchased from Sigma. Primary antibodies against fibronectin (1 : 500), laminin (1 : 200), and collagen I (1 : 500) were added to the samples. After an hour of incubation, samples were washed and incubated with appropriate anti-mouse isotype IgG-Alexa Flour-594 (1 : 500) and anti-rabbit IgG-Alexa Flour-594 (1 : 500) secondary antibodies (Molecular Probes, Invitrogen). After an hour of incubation, samples were washed with 1x PBS and mounted in Vectashield mounting medium containing 4′,6-diamidino-2-phenylindole (DAPI). Images were captured using acoustooptical beam splitter confocal microscope (Leica Microsystems, Germany).

### 2.7. *Ex Vivo* HSPC Expansion

#### 2.7.1. HSPC Expansion on HS-5 Derived ACM

24-well TCP-coated with native or spin coated HS-5 ACM were used for expansion experiments. Serum-deprived intact HS-5 cells were used as a cellular control and normal TCP wells without any matrix or cells were used as negative controls. Magnetically sorted HSPC with >90% CD34^+^ cell enrichment was suspended in Stemline II serum free media (Sigma) containing 50 *μ*g/mL penicillin and streptomycin at a density of 1 × 10^4^ viable cells/mL. The medium was supplemented with growth factors as indicated—thrombopoietin (TPO, 25 ng/mL), stem cell factor (SCF, 25 ng/mL), interleukin-6 (IL-6, 50 ng/mL), and Flt3 ligand (Flt3, 40 ng/mL) (Millipore, USA). 1 mL cell suspension was placed in each well of the 24-well plate in triplicate for each substrate, that is, native ACM, spin coated ACM, TCP, and HS-5 cellular control. All cultures were placed in an incubator with an atmosphere of 5% O_2_, 5% CO_2_, and 95% humidity at 37°C for 8 days, undisturbed and without a media change. After 8 days, the cells were harvested and were processed for immunophenotypic analysis by flow cytometry and functional CFU-c assays.

#### 2.7.2. Flow Cytometry

Flow cytometry for multilineage cell surface markers was performed on HSPC before and after expansion in order to calculate the fold change in the numbers of lineage-specific progenitors. CD34^+^CD45^lo^ phenotype was used for all progenitors committed for hematopoiesis: CD34^+^CD133^+^ and CD34^+^CD38^−^ for cells with an early commitment to granulocyte, erythrocyte, monocyte, and megakaryocyte lineages. 7-Amino-actinomycin D (7-AAD) was added to measure the cell viability. The methodology for HSPC pre- and postexpansion flow cytometry analysis was followed as described elsewhere [15]. Briefly, 2 × 10^4^ HSPC cells (pre- and postexpanded) were added to Fluorescence-Assisted Cell Sorting (FACS) tubes containing 100 *μ*L of PBS/2% FACS solution, and appropriate antibodies and viability dye were added. Stained cells were incubated at RT in the dark for 20 min, followed by washing with 1x PBS containing 2% FCS to remove excess antibody. Finally the cell pellets were resuspended in 300 *μ*L fixative solutions (PBS 2%, FCS 1%, formaldehyde 2 *μ*g/mL, and actinomycin D) and stored at 4°C in the dark. Data acquisition and analysis were performed within three days in FACS Calibur flow cytometer using CellQuest software. Antibodies used in FACS were purchased from Becton Dickinson Biosciences, USA. For determining the mean and standard error values, data from three independent experiments with triplicate samples in every experiment was used. For determining the fold increase in total cell number and lineage-specific cells the calculations were done as follows:

(1) (a) Fold  expansion  of  total  cells=Count  of  total  viable  cells  post-expansionCount  of  total  viable  cells  pre-expansion.

(2) (b) For  fold  expansion  of lineage-specific  cells =Lineage-specific  cell  percentage×Count  of  total  viable  cells  post  expansionLineage-specific  cell  percentage×Count  of  total  viable  cells  pre-expansion.

#### 2.7.3. Colony Forming Unit Assays

Colony forming unit (CFU) assays to evaluate the functional properties of cells grown on the two substrates and control surfaces were done using MethoCult media (H4434) following the standard protocol described elsewhere [15]. Total 3 × 10^3^ viable cells were suspended in the media, plated per well as per the manufacturer's instructions, and incubated for 14 days in 5% CO_2_ and 20% O_2_ at 37°C. CFUs were manually counted under the light microscope following 14 days of incubation. Statistical significance of the variation in CFU numbers was based upon data from three independent experiments. Fold expansion of CFUs in each culture was calculated as explained for marker-based cell expansion. CFUs of granulocyte-macrophage (GM), burst forming units-erythroid (BFU-E), and granulocyte, erythrocyte, monocyte, and macrophage cells (GEMM) were performed with preculture and postculture HSPC cells. Using the following formula, fold change in total and each lineage-specific CFUs was calculated:

(3) Fold  expansion  of  CFU =Post-expansion  CFU  numbersPre-expansion  CFU  numbers.

### 2.8. Statistical Analysis

Experiments were repeated with three different UCB samples. Experimental data were expressed as mean ± standard error (SE). One-way ANOVA was applied to determine the statistical relevance of the total cell and colony expansion on various substrates using Prism 5.0 (GraphPad S) and a value of *p* ≤ 0.05 was considered as significant.

## 3. Results 

### 3.1. ACM Characterization

#### 3.1.1. Phase Contrast Microscopy

**Figure 2:**
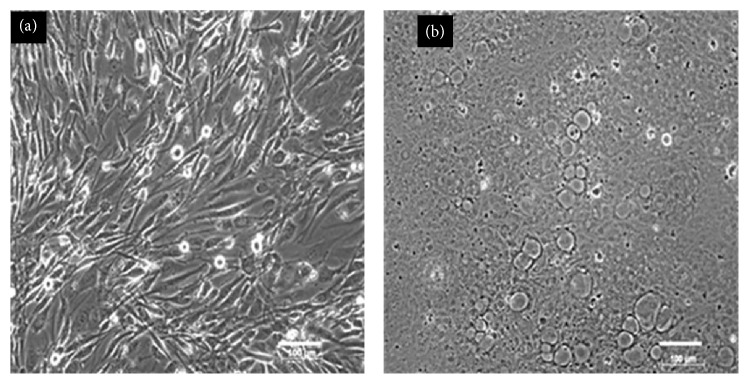
Phase contrast microscope images of HS-5 stromal cells (a) before decellularization and (b) after decellularization (scale bar: 100 *μ*m).

After HS-5 cells had been decellularized by treatment with NH_4_OH, the cell debris was removed leaving behind the ACM. Phase contrast micrographs of HS-5 cells before (Figure 2(a)) and after (Figure 2(b)) NH_4_OH treatment clearly demonstrate the absence of cell nuclei in the decellularized native ACM (Figure 2(b)). The spindle-shaped cells absorb NH_4_OH-containing water. They become round and burst due to excessive accumulation of water leaving behind some cellular components within the ECM. Complete decellularization can be confirmed by the absence of intact nuclei. Hence, decellularization efficiency was further evaluated by staining with DAPI for the absence of intact DNA. The DAPI staining of intact HS-5 cells and decellularized ACM is shown in Supplementary Figure S1.

#### 3.1.2. Scanning Electron Microscopy

**Figure 3:**
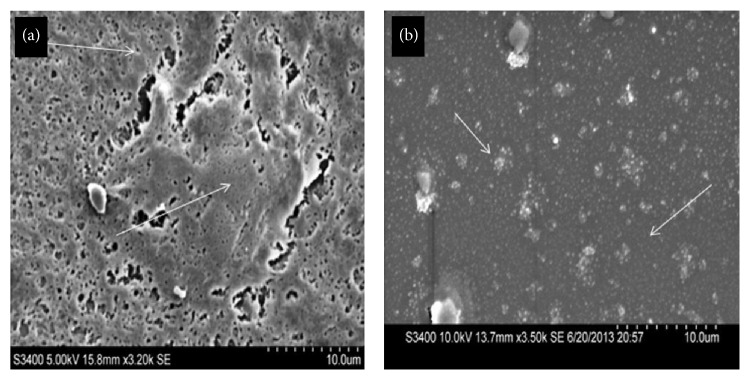
Morphological characterization of native ACM and spin coated ACM. SEM images depicting (a) presence of thick, bulky structures on native ACM and (b) uniform layer of matrix with the presence of subnanosized globular structures on spin coated ACM (scale bar: 10 *μ*m).

SEM analysis of the native and spin coated ACM showed the microdetailing and architecture of the matrix surfaces. Native ACM (Figure 3(a)) exhibit the presence of thick bulky structures, which are the deposited matrix component after decellularization. The porous surface structure is visible as irregular shape measuring few *μ*m in diameter. On the other hand, spin coated ACM (Figure 3(b)) exhibited more uniform layer with the presence of nanosized globular structures on the entire surface.

#### 3.1.3. Atomic Force Microscopy

**Figure 4:**
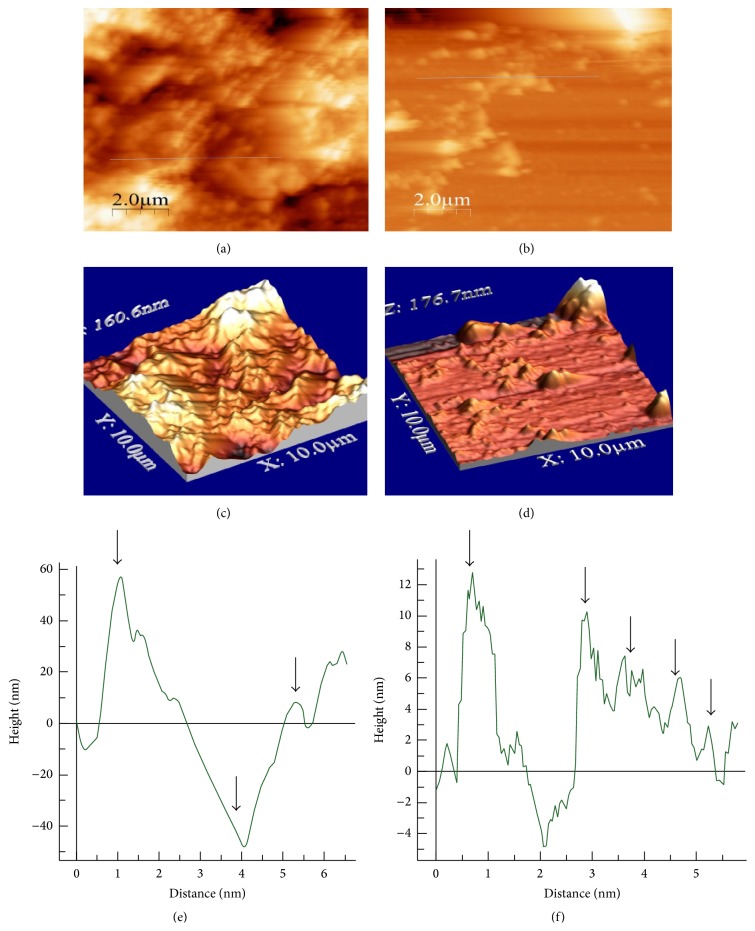
Height variation measurement by 2D and 3D AFM of ACM. (a) 2D AFM of native ACM, (b) 2D AFM of spin coated ACM, (c) 3D AFM of native ACM, (d) 3D AFM of spin coated ACM, (e) line scan to measure the height variations in native ACM, and (f) line scan to measure the height variations in spin coated ACM. Arrows indicate variations in the height of native and spin coated ACM.

AFM analysis of native and spin coated ACM was performed to understand the topographical differences (thickness, roughness, and uniformity) on the surface of the matrices. To analyze the 2D and 3D asymmetry of the coated surfaces, line scan AFM imaging was done for both the substrates. Based on 2D analysis, large variations in surface height and other structural irregularities could be seen on the native ACM (Figure 4(a)) as compared with the spin coated ACM, which was more uniform and symmetrical (Figure 4(b)). The 3D data analysis (Figures 4(c) and 4(d)) showed these differences more clearly as dark and light shades. The units and dimensions of representative line scans of native and spin coated ACM are shown in Figures 4(e) and 4(f). The highest and lowest peaks on native ACM were at +60 nm and −40 nm, respectively (Figure 4(e)), and that for spin coated ACM were at +12 nm and −4 nm, respectively (Figure 4(f)). The high range of difference in the values of surface height suggests that native ACM surface was more irregular than spin coated ACM. The spin coated ACM showed repetition of similar surface height profile in a line scan.

**Figure 5:**
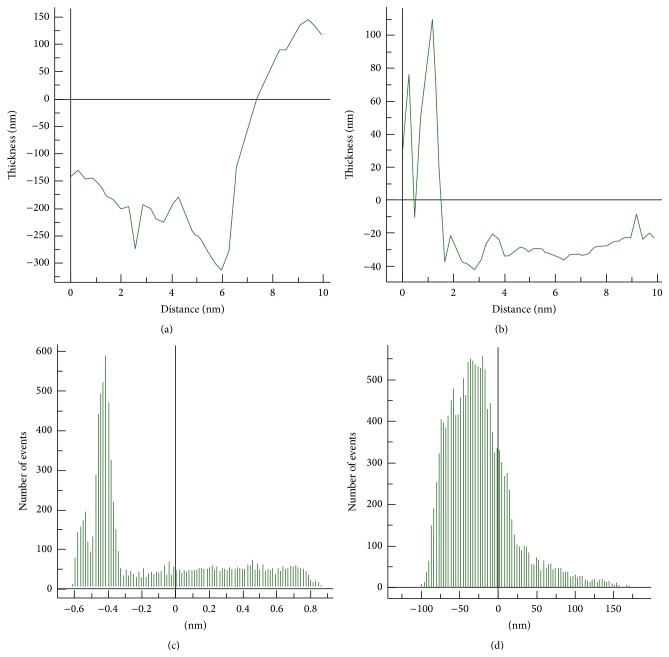
ACM thickness and surface roughness measurement in AFM. (a) Representative figure of Z-scan to measure the thickness of native ACM, (b) Z-scan to measure the thickness of spin coated ACM, (c) representative graph of surface roughness profile in native ACM, and (d) representative graph of surface roughness profile in spin coated ACM.

The thickness of matrix deposited on the native and spin coated ACM was measured by AFM. The average thickness was calculated to be equal to the difference between the scratched and the exposed glass surface and the average matrix deposit level. In native ACM, different levels of thickness were observed to be consistent with the mixed symmetries present on the surface. The average thickness was calculated by scanning random segments in different frames and multiple scans were used for the final calculation in both the substrates. The representative AFM image of the thickness profile of native ACM is shown in Figure 5(a) and that of spin coated ACM is shown in Figure 5(b). The thickness of the native ACM was calculated to be 583 ± 130 nm and that of spin coated ACM was 160 ± 11 nm (Supplementary Table 2). The thickness profile generated by AFM indicated that spin coated ACM are indeed thin film matrices layered uniformly over an area whereas the native ACM are thicker matrices.

For measurement of surface roughness, a total of four scans from random segments of each substrate were used, and the average root mean square roughness of native and spin coated ACM was calculated by the WSxM v4.3 software. The results are shown in Figures 5(c) and 5(d), respectively and, as can be seen, the values were 26 ± 16* Ra* for native ACM and 51.25 ± 3.4* Ra* for spin coated ACM (Figure 5(d)). SEM and AFM analysis of blank coverslips is shown in Figure S2.

### 3.2. Cytochemical Characterization

**Figure 6:**
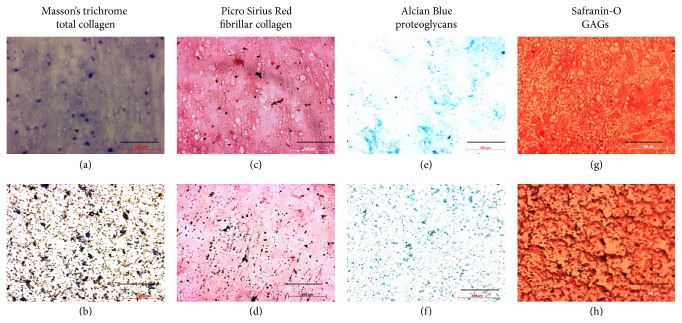
Cytochemical characterization of ACM (“(a), (c), (e), and (g)” indicate native ACM and “(b), (d), (f), and (h)” indicate spin coated ACM). (a) and (b) Masson's trichrome staining of collagen, (c) and (d) Picro Sirius Red staining of fibrillar collagen, (e) and (f) Alcian Blue staining of proteoglycans, (g) and (h) Safranin-O staining of GAGs (scale bar: 100 *μ*m).

Cytochemical staining of substrates showed that both native and spin coated ACM depict similar retention of matrix constituents such as collagen (Figures 6(a) and 6(b)), fibrillar collagen (Figures 6(c) and 6(d)), proteoglycans (Figures 6(e) and 6(f)), and GAGs (Figures 6(g) and 6(h)). In contrast to the native ACM, where the stains are retained by deposited matrix leaving spaces in between, the spin coated ACM exhibit more uniform stain retention. The differences in the physicochemical properties of the substrates did not seem to have affected the qualitative distribution of matrix proteins on the two substrates. Cytochemical staining images for Masson's trichrome, Picro Sirius Red, Alcian Blue, and Safranin-o on blank TCP are shown in Figure S3.

### 3.3. Immunofluorescence Characterization

**Figure 7:**
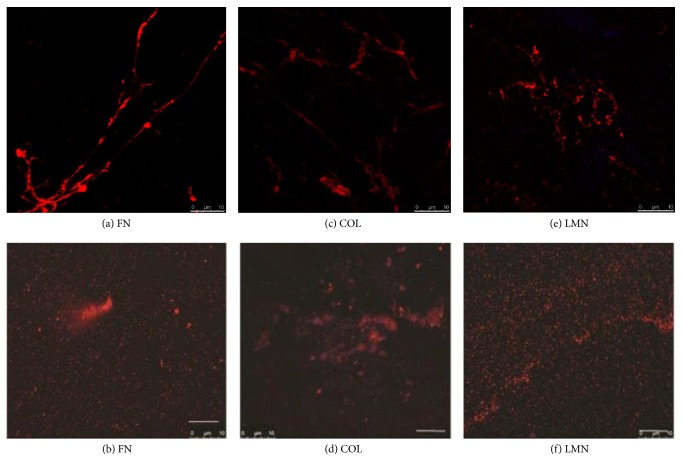
Characterization of native and spin coated ACM by immunostaining (“(a), (c), and (e)” indicate native ACM and “(b), (d), and (f)” indicate spin coated ACM). (a) and (b) Immunostaining of fibronectin, (c) and (d) immunostaining of collagen, and (e) and (f) immunostaining of laminin (scale bar: 10 *μ*m).

Antibody staining of native and spin coated ACM confirmed the presence of all the three major ECM molecules, that is, fibronectin (Figures 7(a) and 7(b)), collagen type I (Figures 7(c) and 7(d)), and laminin (Figures 7(e) and 7(f)) in both the substrates. No nuclear staining (as shown by DAPI staining) or presence of cytoplasmic actin (as shown by Alexa Fluor-488 phalloidin actin staining) could be seen. Native ACM exhibited structural features that were similar to the native stromal structure, whereas the spin coated ACM exhibited uniformly distributed nonfibrous particulate matrix on the surface (Figures 7(e) and 7(f)). Control images of immunofluorescence studies are shown in Figure S4.

### 3.4. HSPC Expansion on ACM

#### 3.4.1. HSPC Cell Morphology

**Figure 8:**
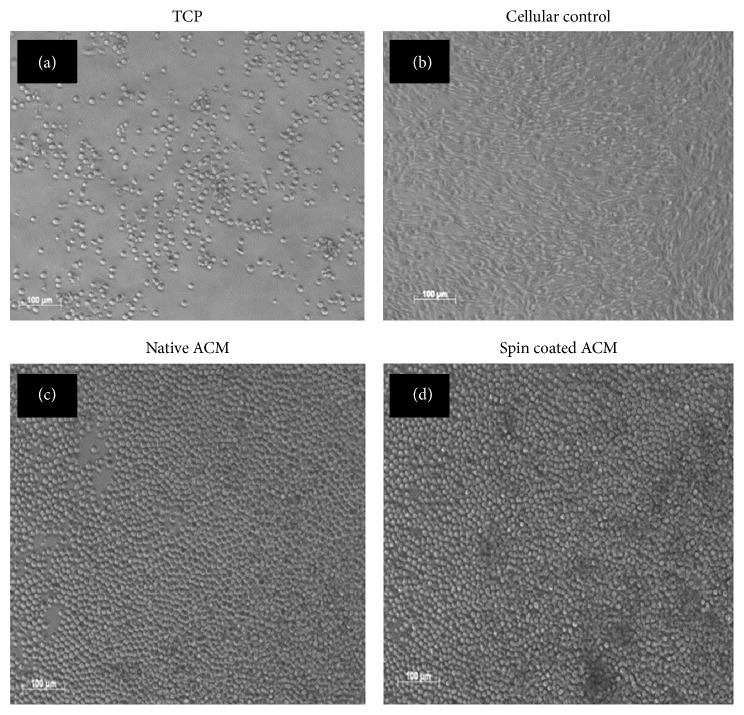
UCB HSPC expansion on various ACM and control surfaces. (a) TCP—*no matrix.* (b) Cellular control (*stromal cells*). (c) Native HS-5 ACM. (d) HS-5 spin coated ACM.

After 8 days of HSPC expansion, images were taken on a light microscope (Axiovert Live cell 200M, Carl Zeiss, Germany). The maximum increase in cell number after 8 days of expansion in culture was observed on spin coated ACM followed by native ACM (Figure 8). HSPC cell morphology was best retained in native and spin coated ACM as compared to the control substrates.

#### 3.4.2. Immunophenotypic Analysis of HSPC Expansion

**Figure 9:**
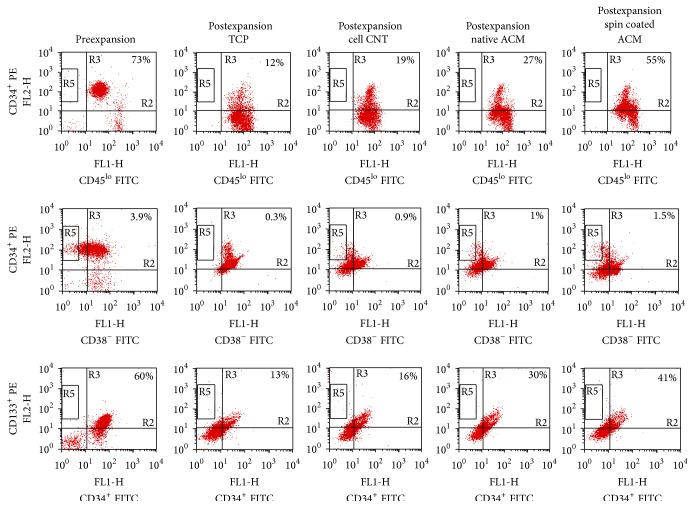
Representative dot plot image of surface marker-based flow cytometry assay indicating the percentage of CD34^+^CD45^lo^, CD34^+^CD38^−^, and CD34^+^CD133^+^ cell subsets in preexpanded cells and cells expanded on TCP, on cellular control, on native ACM, and on spin coated ACM.

The flow cytometry data was obtained from three different UCB derived HSPC and the average cell viability and percentage of each lineage-bearing cell were calculated. The corresponding dot plots for CD34^+^CD45^lo^, CD34^+^CD38^−^, and CD34^+^CD133^+^ cells, before and after expansion on the substrates, have been shown in Figure 9. Fold expansion was calculated according to the formula discussed earlier in Section 2.

**Figure 10:**
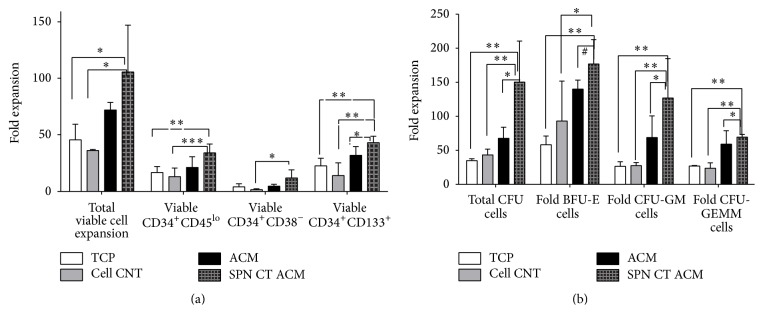
(a) Immunophenotypic analysis for measurement of fold increase in UCB HSPC of different lineages after expansion on native ACM, spin coated ACM, TCP, and cellular control. The graphs represented in the figure are the means of three independent experiments. (^*∗*^ *p* ≤ 0.05, ^*∗∗*^ *p* ≤ 0.005, ^*∗∗∗*^ *p* ≤ 0.001, ^#^no significance, and *n* = 3). (b) Functional analysis of fold expansion of UCB HSPC by colony forming unit assay for CFU-GM, CFU-GEMM, and BFU-E as progenitor cell population on native ACM, spin coated ACM, TCP, and cellular control. (^*∗*^ *p* ≤ 0.05, ^*∗∗*^ *p* ≤ 0.005, ^#^no significance, and *n* = 3).

Our results indicate that spin coated ACM could better support the expansion of CD34^+^CD133^+^ bearing progenitors 44-fold on spin coated ACM* versus* 35-fold on native ACM in comparison to the controls (Figure 10(a)). Spin coated substrates were also good for expanding committed HSPC (CD34^+^CD45^lo^), as compared to TCP and cellular control but not for undifferentiated CD34^+^CD38^−^ progenitors (Figure 10(a)).

#### 3.4.3. Colony Forming Cells Analysis of HSPC Expansion

CFU-c data were obtained from three independent experiments of HSPC expansion on ACM substrates and controls. Comparison of results of expansion between different substrates is represented in Figure 10(b). Although expansion of all cell types (CFU-GM, BFU-E, CFU-GEMM, and total viable cells) was observed on both ACM, the expansion of BFU-E (170-fold) was efficient in spin coated ACM as compared to the TCP and cellular controls. CFU-GM (120-fold) and CFU-GEMM (70-fold) were more efficient and significant on spin coated ACM as compared to the native ACM, TCP, and cellular controls (Figure 10(b)).

## 4. Discussion

The discovery of HSPC and its application for restoration of normal hematopoiesis in malignant and nonmalignant, haematological disorders [2] has been a landmark in the field of cell transplantation. Very often, the success of this treatment is limited due to the low numbers of repopulating stem cells in the graft. This deficiency can be improved by the expansion of HSPC* ex vivo* so that the requirement of a large number of uncommitted primitive cells to repopulate the BM and restoration of normal hematopoiesis is met.

The fact that the hematopoietic niche regulates HSC self-renewal and proliferation* in vivo* has inspired researchers to recreate niche like* in vitro* conditions for achieving lineage-specific HSPC growth. The hematopoietic niche in BM is a complex biological milieu; it comprises several cellular and molecular components, soluble growth factors, and nonsoluble ECM [6]. Both the cellular and extracellular counterparts of the hematopoietic niches have been investigated extensively to understand their stem cell regulatory activities and properties [16]. It has been demonstrated that niche components can regulate adhesion, proliferation, differentiation, and survival of HSPC in the BM [6]. ECM molecules anchor growth factors and they provide mechanical strength to the niche for facilitating interactions between other niche components [17].

Earlier studies have focussed on individual ECM components for HSPC expansion, which do not represent the* in vivo* complexity of the hematopoietic niche [18–20]. For example, a cocktail of different ECM molecules did not give significant* ex vivo* expansion of HSPC probably because they did not provide all the required extracellular signals necessary for HSPC expansion* in vivo* [11]. Our study has succeeded on this account because we have used the entire ACM, which potentially contain all ECM molecules along with associated growth factors and other regulatory molecules that are required for HSPC expansion. In this report, we have demonstrated that ACM derived from human BM stromal cell line, both in native or spin coated forms, could efficiently support* ex vivo* HSPC expansion. We have also shown that these ACM are enriched in fibronectin, laminin, collagen, and ECM-associated growth factors as observed in the ECM of* in vivo* hematopoietic niches [21, 22]. Previous studies have demonstrated that HSPC can enhance their functions by directly binding to ECM molecules; for example, fibronectin enhances CFU-GM and BFU-E progenitor cell growth* ex vivo* by binding to *α*4 and *α*5 integrins on these cells [23]; similarly immobilized fibronectin used as 3D scaffolds [24] or coatings and fibrillar collagen-I associated with glycophosphoprotein and osteopontin support the expansion of UCB derived HSPC [13]. We have demonstrated the enhanced expansion of HSPC on HS-5 derived ACM. Our results suggest that HS-5 derived ACM mimic the* in vivo *properties of the vascular niche in the BM. To our knowledge, this is the first report on the use of ACM derived from human stromal cells that significantly supports the expansion of UCB derived HSPC. An additional advantage of our results is that HS-5 derived ACM can be used in the clinical studies where xenogeneic substrates are not permitted due to immunological considerations. We also demonstrate that, along with biochemical properties, the topographical and physical features also play a critical role in HSPC growth and survival. We could observe an improvement in the lineage-specific HSPC expansion of committed progenitor cells population on spin coated HS-5 ACM as compared to native ACM. One possible explanation is that the topographical differences in surface roughness, thickness, and the uniform nanoglobular surface architecture of the substrates could be responsible for this functional improvement. With the spin coating, the matrix proteins are more uniformly distributed which probably helps in improving the total surface area of interaction of the cells with the matrix components. The thickness of the matrix is also known to affect the fate of the cells [25]. It has been shown that thinner matrices having higher elastic modulus and stiffness can better mimic the rigid bone microenvironment [25]. The nanoglobular surface features of tissue-engineered substrates are also known to support better osteogenic functions. Surface microtopography and roughness have also been reported to play an important role in the differentiation of MSCs [26] and embryonic stem cells [27] and in the adhesion, spreading, proliferation, and differentiation of osteogenic cells [28].

Based on our results, we propose that topographical features of spin coated ACM that are thinner (3.5-fold) and more rough (2-fold) than native ACM can support a better expansion of committed progenitor of hematopoietic cell populations. These topographical features such as uniformity, stiffness, and roughness of the spin coated ACM could have contributed to the improved expansion in spin coated ACM. Our data confirm that an optimum substrate topography and roughness are necessary for achieving enhanced availability of the nutritive medium and growth factors to the cells, and, therefore, such surfaces are better than others for supporting all cell functions.

Recent reviews also highlight the use of various technologies in the development of efficient biomaterials, using one or more ECM components for tissue engineering applications [29–31]. Such advancements in biomaterial designing can be applied to extract maximum potential of human tissue-specific ACM without compromising the functional properties of individual ECM components to get enhanced expansion of lineage-specific HSPC cells* ex vivo*.

## 5. Conclusion

Our report describes a simple and novel method to prepare native and spin coated ACM that mimic functional properties of the hematopoietic niche in human BM. ACM prepared by our method can provide better understanding of physicochemical and topographical properties of hematopoietic niches and reveal novel regulatory roles of soluble and ECM bound signalling molecules. Since the matrix can be prepared in advance and stored, this can be used as a simple coating material with a known concentration, thereby minimizing the variation that is seen when using the feeders cells as culture system. The native and spin coated ACM can be stored in sterile conditions for a longer period as compared to conventional biological substrates. Our methodology also provides a general protocol to prepare ACM from other cell sources (e.g., embryonic fibroblasts and foreskin fibroblasts) and replace their use as feeder layers to minimise variations in various tissue engineering experimental setups.

## Supplementary Material

The efficiency of decellularization of the HS-5 stromal cells with 0.02 M NH_4_OH treatment was analyzed in phase microscope; matrices were stained to confirm the complete removal of nuclear material using DAPI, also, DNA content in HS-5 stromal cells and decellularized ACM was quantified (Figure S1). Table S1: Total matrix protein estimation after decellularization. The blank glass coverslips with no cells, native or spin coated matrix was taken as negative control for the surface topographical analysis in AFM (Figure S2). Table S2: The average thickness of native and spin coated matrices measured in AFM. The native and spin coated matrices were also characterized by cytochemical and immunofluorescence stainings for matrix proteins (Figures S3 and S4: Representative images of negative controls).
