# Supplementary material for: Enhanced Ex Vivo Expansion of Human Hematopoietic Progenitors on Native and Spin Coated Acellular Matrices Prepared from Bone Marrow Stromal Cells
Source: Stem Cells Int. 2016 Feb 14;2016:7231567. doi: 10.1155/2016/7231567 (PMC4769778; doi:10.1155/2016/7231567)
Supplement: Supplementary file 1 — The efficiency of decellularization of the HS-5 stromal cells with 0.02 M NH4OH treatment was analyzed in phase microscope; matrices were stained to confirm the complete removal of nuclear material using DAPI, also, DNA content in HS-5 stromal cells and decellularized ACM was quantified (Figure S1). Table S1: Total matrix protein estimation after decellularization. The blank glass coverslips with no cells, native or spin coated matrix was taken as negative control for the surface topographical analysis in AFM (Figure S2). Table S2: The average thickness of native and spin coated matrices measured in AFM. The native and spin coated matrices were also characterized by cytochemical and immunofluorescence stainings for matrix proteins (Figures S3 and S4: Representative images of negative controls). [file 7231567.f1.pdf]

Supplementary information

Matrix Characterization

Cytochemistry

Alcian blue staining for ECM-bound glycosaminoglycans (GAGs) before and after decellularization was done as follows. Cultures were fixed in 4% formaldehyde and permeabilized with 0.5% Triton-X100 for 30 min, washed two times with PBS and rinsed in 3% acetic acid (pH 2.5) for five minutes to equilibrate the pH. Samples were then incubated with 1% alcian blue for 2 h, washed in 3% acetic acid, followed by 3% acetic acid/50% ethanol, and two water wash. Presence of GAGs was verified by the accumulation of blue stain as observed under phase microscope with 10X magnification.

The retention of a collagenous matrix following the NH_4OH treatment was verified by positive staining for collagen by light green. Briefly, samples were washed three times in PBS and fixed in ice cold 75% ethanol for 10 min, incubated with light green stain at 25°C for 30 min on orbital shaker before being washed with ddH_2O until no leaching was detected. This was followed by 75% ethanol wash to remove non-specific dye and then dried at 25°C and were examined for the presence of greenish-blue stain under phase microscope with 10X magnification.

HSPC expansion

Colony Forming Assays

Colony forming assays were preformed for all cell samples, as triplicate cultures in Methocult media (H4434) in 12 well plates, to assess the numbers of colony forming units (CFUs) for granulocyte, erythrocyte, macrophage, megakaryocyte common precursors (CFU-GEMM), granulocyte/macrophage common precursors (CFU-GM), and burst forming units (BFU-E) for pure erythroid precursors. 2 X 10^3 viable cells were suspended in the media, plated per well as per the manufacturer's instructions and incubated for 14 days in 5% CO_2 and 20% O_2 at 37ºC. CFUs were manually counted under light microscope with 5X magnification following 14 days of incubation. Statistical significance of the variation in CFU numbers was based upon data from three independent experiments. Fold expansion of CFUs in each culture was calculated as explained for marker-based cell expansion.

Supplementary figures

Figure S1-

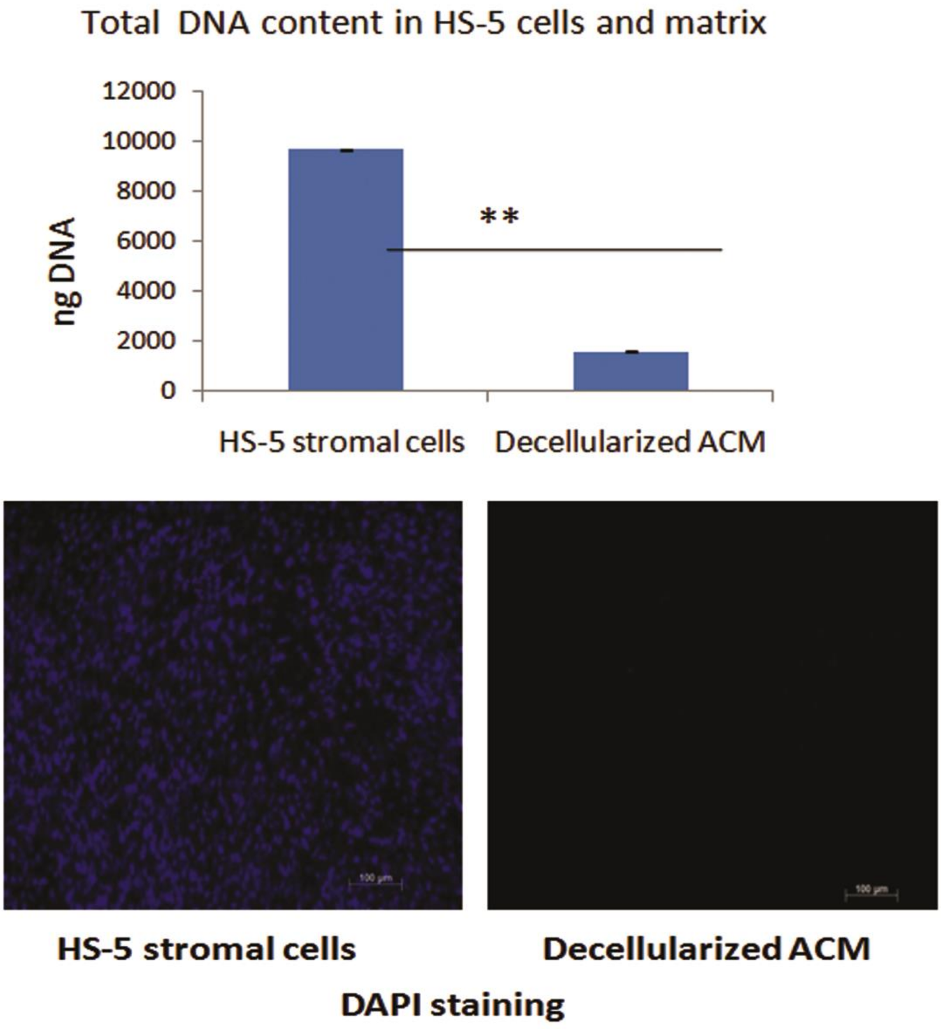

Figure S1. A) DNA quantification in HS-5 stromal cells and decellularized ACM. (**p<0.01). B) Nuclear staining with DAPI– left image depicts HS-5 stromal cells, right image depicts decellularized ACM.

Figure S2

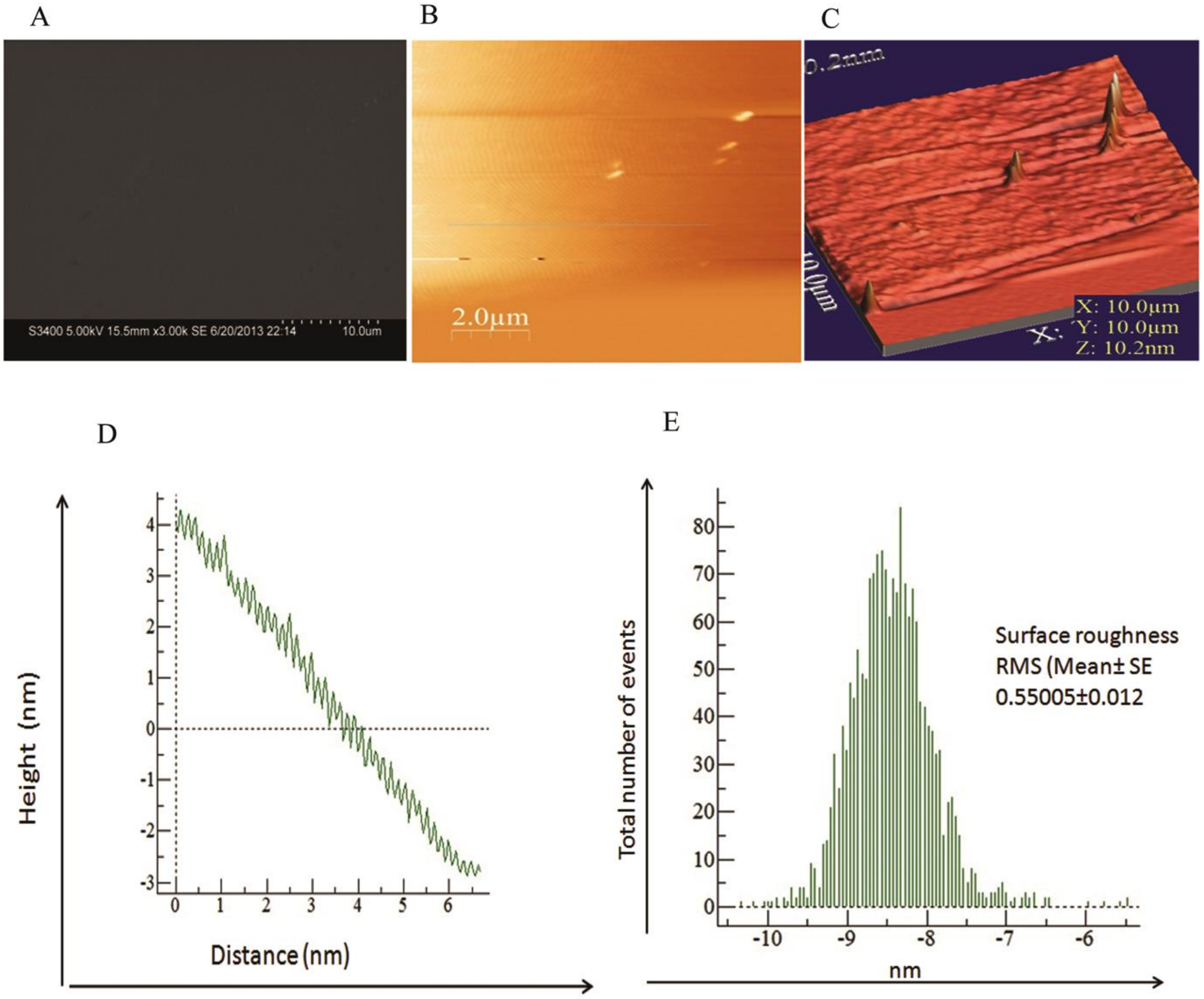

Figure S2. SEM and AFM analysis of blank coverslip. A) SEM image, B) 2D AFM image, C) 3D AFM image, D) Analysis of height variation in blank coverslip by AFM line scan, E) Surface roughness analysis of blank coverslip in AFM.

Figure S3

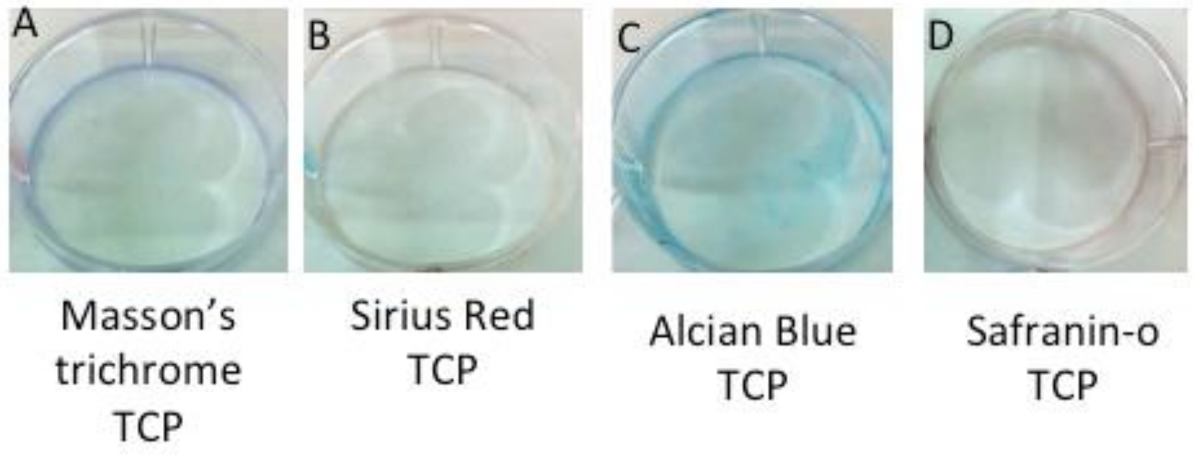

Figure S3- Cytochemical stainings on blank tissue culture plates as negative controls –A) Masson's trichrome , B) Sirius Red, C) Alcian Blue D) Safranin-o

Figure S4

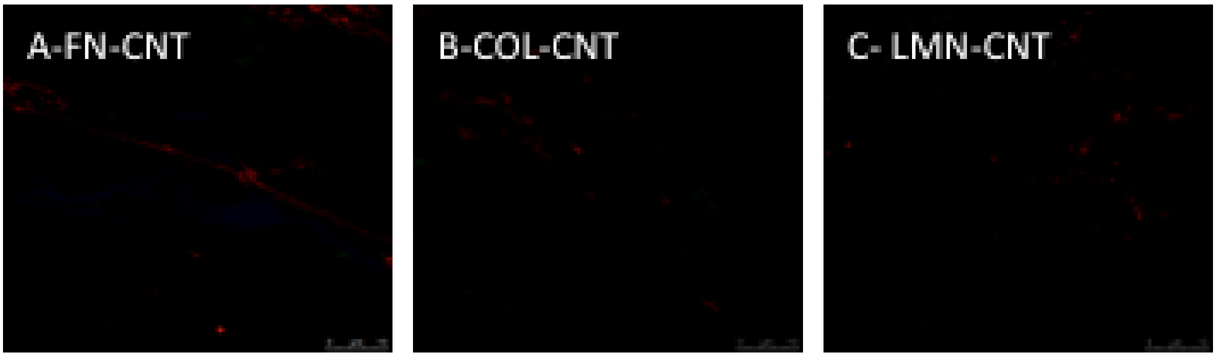

Figure S4-Negative controls – Immunofluorescnce stanings A) Fibronectin control B) Collagen control C) Laminin control stainings

Supplementary Table

Table S1. Total protein estimation in decellularized ACM (each one well of 24 well tissue culture plate)

	**1**	**2**	**3**	Avg
µg/per well	44.6	58.8	67.2	56
STDEV	±9.2	±2.7	±15.8	±9

Table S2. Matrix surface thickness measured by AFM in native ACM and spin coated ACM

	Matrix Thickness Native ACM (nm)	Matrix Thickness SPIN COAT ACM (nm)
1	600	140
2	800	180
3	350	160
Avg (Mean ±SE)	583 ± 130	160 ±11
